# Ultrasound‐Guided Nerve Block: A Potential Intervention for Drug‐Refractory Allergic Rhinitis

**DOI:** 10.1002/ccr3.71669

**Published:** 2025-12-15

**Authors:** Ruimeng Tian, Sisi Nie, Changmei Kou, Meizu Wang, Shiyi Pu, Zhihai Li

**Affiliations:** ^1^ The Affiliated Anning First People's Hospital of Kunming University of Science and Technology Kunming China

**Keywords:** allergic rhinitis, image‐guided intervention, sphenopalatine ganglion block, ultrasound‐guided

## Abstract

Ultrasound‐guided sphenopalatine ganglion block presents a promising, minimally invasive intervention for patients with drug‐refractory allergic rhinitis. This case demonstrates its potential to achieve significant symptom relief and quality‐of‐life improvement with a favorable safety profile, offering an alternative when conventional pharmacotherapy fails.

## 1 Introduction

Allergic rhinitis (AR), a prevalent chronic inflammatory disorder of the nasal mucosa, has emerged as a significant worldwide health concern. Current epidemiological data reveal a steadily increasing global prevalence, now affecting approximately 18.1% of the population [1]. This upward trend correlates strongly with modern environmental changes and evolving lifestyle patterns. The pathophysiology of AR involves IgE‐mediated type I hypersensitivity reactions. Exposure to common aeroallergens—including pollen, dust mites, and animal dander—triggers an exaggerated Th 2 immune response in sensitized individuals. This cascade leads to nasal mucosal inflammation and the classic symptom tetrad: persistent nasal congestion, profuse rhinorrhea, paroxysmal sneezing, and intense nasal pruritus [2].

The clinical implications of AR extend far beyond nasal symptoms. The condition significantly impairs quality of life metrics, frequently causing sleep architecture disturbances and cognitive performance deficits. Importantly, AR demonstrates a well‐established comorbidity with asthma and other atopic conditions. The chronic, relapsing nature of the disease necessitates prolonged pharmacotherapy, creating substantial socioeconomic burdens through both direct healthcare costs and indirect productivity losses [3].

In traditional Chinese medicine, acupuncture therapy has demonstrated efficacy in alleviating rhinitis symptoms through precise stimulation of the sphenopalatine acupoint [4, 5]. The therapeutic mechanism involves modulation of the vagus nerve–macrophage axis, which exerts potent anti‐inflammatory effects [6]. However, the efficacy of acupuncture is highly dependent on the practitioner's expertise, the precise localization of acupoints, and the skill involved in needle insertion. Considerable variation exists in acupoint selection, treatment frequency, and the total number of sessions across different therapeutic approaches. Furthermore, as acupuncture does not involve pharmaceutical agents, its therapeutic effects tend to be of relatively short duration, often necessitating a strict and prolonged treatment regimen [7, 8].

With recent advancements in interventional techniques, ultrasound‐guided nerve blockade has emerged as a promising therapeutic modality for various clinical conditions. Building upon the principles of acupoint stimulation, we present a novel application of ultrasound‐guided sphenopalatine ganglion (SPG) blockade for symptom relief in allergic rhinitis (AR).

## 2 Case History

A 38‐year‐old female presented to our institution with a 12‐year history of poorly controlled AR. The patient's symptoms initially developed following her relocation to Yunnan Province, characterized by morning nasal pruritus, paroxysmal sneezing, and watery rhinorrhea lasting 20–40 min. Clinical progression was noted in the second year, with symptom exacerbation during spring seasons, including new‐onset ocular symptoms (pruritus, photophobia, and epiphora), increased frequency (> 4 episodes weekly), and prolonged duration, particularly following exposure to dust and pollen. These symptoms significantly impacted her quality of life and occupational performance.

Diagnostic evaluation confirmed persistent allergic rhinitis (PAR) with sensitization to multiple allergens, including pollen, animal dander, tobacco smoke, and dust mites. Initial therapeutic management consisted of Tongqiao Biyan tablets (1.5 mg tid), intranasal budesonide spray, and saline nasal irrigation, which provided partial symptomatic relief without achieving complete remission.

Five years ago, the patient was transferred to another job, and the work pressure increased. The clinical deterioration was characterized by worsening rhinitis symptoms, along with systemic manifestations such as fatigue and sleep disturbances. Initially, to manage her symptoms, she was prescribed diazepam (2.5 mg hs po) for sleep assistance, along with olopatadine eye drops for ocular symptoms and oral loratadine (10 mg qd po). Subsequently, as the symptoms worsened, the doctor prescribed antihistamines and steroid drugs for systemic treatment. However, this patient often failed to follow the doctor's instructions in taking the medicine regularly; the symptoms of her rhinitis were not well controlled. She experienced significant disruptions in daily functioning, prompting her to seek further evaluation and treatment in our department.

After multiple pharmacological interventions failed to achieve suboptimal symptom control, and given that the patient's polysensitivity precluded the feasibility of a long‐term, meticulous allergen‐specific therapy due to financial and occupational constraints, we decided to implement an ultrasound‐guided sphenopalatine ganglion block to alleviate and manage the patient's rhinitis symptoms.

## 3 Investigations and Treatment

**FIGURE 1:**
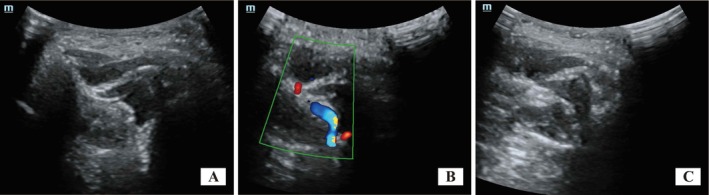
Ultrasound‐guided SPG block. (A) Anatomical view of the sphenopalatine fossa (white arrow). (B) Position of the maxillary artery (red arrow). (C) Ultrasound image of needle insertion (yellow arrow).

The patient underwent three sessions of ultrasound‐guided SPG block with a 7‐day interval between sessions. The mandibular condyle was first found below the zygomatic arch using a low‐frequency linear array probe (3–5 MHz), and then the probe was moved towards the nasal side to reveal the pterygopalatine fossa, in which the sphenopalatine ganglion was located. The depth of the sphenopalatine ganglion was adjusted to further clearly show the position, shape, and surrounding blood vessels, nerves, and other structures (Figure 1A). The needle was inserted from the cheek in front of the tragus, at an angle of 60°–80° to the face, and the needle was punctured in the direction of the pterygopalatine fossa, avoiding important blood vessels, nerves, and other tissue structures as much as possible (Figure 1B). Under real‐time ultrasound monitoring, the position of the puncture needle was closely observed. Dilute lidocaine hydrochloride (5 mL: 0.1 g), compound betamethasone (1 mL: 5 mg diphenthanolone betamethasone + 2 mg betamethasone phosphate sodium), and vitamin B12 (2 mL: 0.5 mg) were mixed with normal saline to a total volume of 15 mL [9]. When the puncture needle reached the pterygopalatine fossa and was close to the sphenopalatine ganglion, 5 mL block solution was injected (Figure 1C).

The patient was asked to fill out the Total Nasal Symptoms Score (TNSS), the Total Non‐Nasal Symptoms Score (TNNSS), and the Rhinoconjunctivitis Quality of Life Questionnaire (RQLQ) questionnaires before the first injection (week 1), before the second injection (week 2), before the third injection (week 3), and 1 month after the third injection (week 6). The patient signed the consent form for this case report to allow publication of her care. The symptoms of rhinitis were scored.

This study was conducted in accordance with the Declaration of Helsinki and approved by the Ethics Committee. Written informed consent was obtained from the patient(s) for the publication of this case report and any accompanying images.

## 4 Conclusion and Results

The patient demonstrated significant clinical improvement following ultrasound‐ guided sphenopalatine ganglion blockade for allergic rhinitis, with objective evidence of reduced symptom scores (Table 1) and enhanced quality of life metrics (Table 2).

**TABLE 1:** TNSS and TNNSS scores of the patient in each treatment cycle.

	Week 1	Week 2	Week 3	Week 6
TNSS				
Stuffy nose	3	3	2	1
Runny nose	2	2	1	0
Itchy nose	3	3	2	1
Sneeze	3	2	1	1
Total	11	10	6	3
TNNSS				
Itchy eyes	3	2	1	1
Watery eyes	2	1	0	0
Itchy throat	1	0	0	0
Itchy ear	0	0	0	0
Cough	0	1	0	0
Total	6	4	1	1

*Note:* TNSS score of 0–2 indicates mild rhinitis; score of 3–6 indicates moderate rhinitis; and score of 7–12 indicates severe rhinitis. TNNSS score of ≥ 4 points signifies prominent non‐nasal symptoms.

**TABLE 2:** RQLQ scores of the patient in each treatment cycle.

	Week 1	Week 2	Week 3	Week 6
Rhinitis				
Stuffy nose	5	3	3	0
Runny nose	4	3	1	0
Itchy nose	5	4	2	2
Sneeze	4	3	2	1
Dimensional score	4.50	3.25	2.00	0.75
Eye symptoms				
Itchy eyes	4	3	1	1
Watery eyes	4	2	1	1
Eye imitation	2	2	0	0
Tired eyes	3	1	1	0
Dimensional score	3.75	2.00	0.75	0.50
Other symptoms				
Headache	3	2	1	1
Fatigue	4	3	1	1
Cough	1	2	1	0
Inability to focus	3	3	2	1
Dimensional score	2.75	2.50	1.25	0.75
Restrictions on activity				
Impact on housework	1	2	0	0
Affected activity	2	2	1	1
Limitation of outdoor activities	3	3	2	2
Work interrupted	2	2	1	1
Dimensional score	2.00	2.25	1.00	1.00
Sleep				
Difficulty sleeping	5	5	3	2
Do not sleep deeply	5	3	1	1
Dreams	2	2	2	1
Disturbed sleep	4	3	2	1
Dimensional score	4.00	3.25	2.00	1.25
Social functioning				
Annoyed about having to carry hanky & blowing nose	4	4	2	0
Loss of confidence	2	1	1	1
Reduced meetings with others	4	3	1	1
Don't feel like going out	0	0	0	0
Dimensional score	2.25	2.00	1.00	0.75
Emotions				
Anxious/worried	1	1	1	1
Frustrated	0	0	0	0
Fidgety	3	2	1	1
Annoyed with self	0	0	0	0
Dimensional score	1.00	0.75	0.50	0.50

*Note:* Dimensional score = mean score for each dimension. Score of 0 indicates not affected at all; score of 1 indicates minimal effect; Score of 2 indicates slight impact; Score of 3 indicates moderate impact; Score of 4 indicates severe impact; Score of 5 indicates severe impact; Score of 6 indicates extremely high impact.

Progressive symptom relief was observed after each treatment session, with two treatment cycles yielding substantial control of systemic manifestations including resolution of sleep disturbances and fatigue. One month after the last treatment, the patient was followed up again. The results of the system scoring showed that the symptoms of rhinitis had been basically improved, only mild nasal congestion and sneezing in the morning, and the conjunctival symptoms and systemic symptoms were basically controlled (Compared to the initial assessment, TNSS reduction > 50%, TNNSS improvement > 40%), and the patient reported that he no longer took drugs to help him sleep, and the quality of work and life had been significantly improved (Compared to the initial assessment, RQLQ improvement > 0.5 points).

This clinical observation suggests that multiple courses of ultrasound‐guided repeated sphenopalatine ganglion block may be an effective method for the treatment of allergic rhinitis, especially for the patient with poor drug control, unable to adhere to drug treatment for a long time, and difficult desensitization treatment, with an excellent safety profile and no observed adverse events throughout the treatment course.

## 5 Discussion

Chronic rhinitis represents a significant global health concern, affecting approximately 40% of the general population. Of these cases, AR constitutes the predominant subtype, with epidemiological studies demonstrating a persistent upward trend in its worldwide prevalence [10]. This condition, alternatively termed hay fever or allergic rhinoconjunctivitis in medical literature, presents with a characteristic symptom complex. The classic nasal triad of obstruction, rhinorrhea, and paroxysmal sneezing is frequently accompanied by ocular manifestations including pruritus, epiphora, and visual disturbances. In more severe presentations, patients may experience systemic involvement characterized by fatigue, sleep fragmentation, and cephalgia, collectively contributing to substantial impairment in quality of life [11].

The primary therapeutic objective in persistent allergic rhinitis (PAR) management is effective symptom control. Current treatment modalities encompass a spectrum of interventions including allergen avoidance strategies, nasal saline irrigation, oral antihistamines, intranasal corticosteroids, combination intranasal corticosteroid/antistamine sprays, leukotriene receptor antagonists, and allergen immunotherapy [3, 10]. However, clinical challenges persist as 10%–20% of patients with PAR demonstrate suboptimal response to pharmacotherapy. The chronic nature of the condition often leads to disease adaptation in some patients, resulting in poor adherence to long‐term medication regimens (Perception and control of allergic rhinitis in primary care). Furthermore, documented adverse effects associated with glucocorticoids, antihistamines, and antileukotrienes present additional therapeutic limitations. Compounding these challenges is the frequent occurrence of polysensitization in AR patients, coupled with the limited clinical availability of comprehensive allergen preparations for immunotherapy. Consequently, a significant proportion of patients continue to experience disease burden despite available treatments [10].

The pathogenesis of rhinitis symptoms involves neural transmission through the sphenopalatine and nasal nerves. SPG, a key parasynaptic structure of the sphenopalatine nerve located in the pterygopalatine fossa, receives parasympathetic fibers from the facial nerve via the greater superficial petrosal nerve and provides autonomic innervation to the lacrimal gland, nasal mucosa, and palatal regions [3, 7]. SPG activation mediates nasal mucosal edema, hypersecretion, and sneezing reflexes through neurogenic inflammatory pathways.

Emerging clinical evidence supports the therapeutic potential of SPG modulation. While SPG blockade has demonstrated efficacy in migraine management and is gaining recognition for secondary headache treatment [12, 13]. In addition, studies in traditional Chinese medicine have shown that SPG can effectively control the symptoms of rhinitis through acupuncture [4, 5, 6, 14]. However, mechanistic evidence for blockade efficacy in rhinitis requires further validation.

Based on this neuroanatomical and clinical evidence, we hypothesize that targeted SPG blockade may represent a viable therapeutic approach for AR symptom control, warranting systematic investigation to establish its clinical utility and mechanistic basis.

In recent years, the technique of ultrasound‐guided nerve block has evolved and is increasingly used to treat multisystem diseases or for analgesia by blocking tiny nerves [15]. In this case, we configured the blocking fluid and injected it into the SPG. Through the use of ultrasound exploration and real‐time monitoring of the position of the puncture needle, the safety of this treatment method is greatly improved, and the intraoperative injury of nerves and blood vessels is effectively avoided, and the risk of postoperative paralysis, motor dysfunction, and bleeding is reduced. At the same time, this method can effectively control the dosage of drugs and avoid the adverse reactions such as headache and dizziness after ganglion block. In addition, by injecting blocking drugs directly into SPG, the absorption of drugs in the nasopharyngeal mucosa can be effectively avoided, so as to ensure the effectiveness of drug dose and achieve the effect of blocking SPG more effectively [16].

TNSS, TNNSS, and RQLQ were confirmed to be significantly correlated with rhinitis symptoms [17, 18, 19]. In this study, TNSS, TNNSS, and RQLQ were used to assess the symptoms of rhinitis in the patient. During this treatment, we observed the symptoms of rhinitis in the patient after the end of each course of treatment to evaluate the therapeutic effect. In this case, TNSS, TNNSS, and RQLQ scores showed that the symptoms of rhinitis were improved after the first treatment, and the scores of runny nose, nasal itching, and sneezing were decreased. After the last treatment, the systemic symptoms of the patient were basically controlled, and the sleep quality was significantly improved. There were no adverse reactions such as bleeding, headache, and paresthesia during the treatment.

We speculate that the effective reason for this treatment is that it can relieve the symptoms of nasal congestion and rhinorrhea by temporarily blocking parasympathetic nerve conduction, reducing the release of acetylcholine, inhibiting vasodilatation and gland secretion, and regulating the release of neuropeptides: inhibiting the release of pro‐inflammatory neuropeptides such as substance P and calcitonin gene‐related peptide, and reducing neurogenic inflammation. Repetitive block may reduce autonomic hyperresponsiveness through neuroplasticity changes, so as to achieve long‐term relief of patients' symptoms.

The clinical efficacy of this intervention appears to be mediated through a dual mechanism involving both immediate pharmacological effects and long‐term neural adaptations. By temporarily blocking parasympathetic transmission, the treatment reduces acetylcholine release, thereby decreasing glandular secretion and vasodilation while simultaneously modulating neuropeptide release (particularly substance P and CGRP) to attenuate neurogenic inflammation. Furthermore, repeated blocks induce neuroplastic changes that progressively reduce autonomic hyperresponsiveness, leading to sustained symptom relief through gradual recalibration of maladaptive neural circuits involved in rhinitis pathophysiology.

In this scenario, we remained concerned about the patient's adherence to the treatment regimen. Fortunately, the patient consistently attended all scheduled hospital visits for treatment throughout each cycle and actively participated in completing the questionnaires as well as subsequent follow‐ups. The dosage and duration of the blocker administration were based on an empirical protocol. Additionally, a systematic investigation to validate this research has not yet been conducted.

Adverse Events and Complications: Adverse events associated with the procedure primarily included injection site discomfort or pain, minor bleeding or hematoma formation, and transient edema or swelling. Symptoms related to the nerve block itself comprised transient numbness within the maxillary nerve distribution (including the cheek, upper lip, hard palate, and soft palate), a sensation of heaviness or pressure in the deep facial and retro‐orbital region, self‐limited dysgeusia, and reflexive lacrimation on the ipsilateral side. More serious complications, such as local anesthetic toxicity, vascular injury, or neurological injury, were rare. The utilization of ultrasound guidance significantly minimized the incidence of these adverse events by enabling real‐time visualization of the needle path and adjacent anatomical structures.

Ultrasound‐guided sphenopalatine ganglion block offers a novel, targeted intervention for the management of AR. For patients with drug intolerance, poor medication adherence, or contraindications to pharmacotherapy, this minimally invasive neuromodulation technique not only overcomes the limitations of conventional drug treatments but also aligns with the principle of personalized medicine. Current clinical evidence indicates significant short‐term efficacy and favorable safety profiles; however, long‐term effectiveness and the underlying mechanisms require further validation through large‐scale randomized controlled trials. The dissemination and implementation of this technology may provide innovative insights into the clinical management of AR.

## Author Contributions

**Ruimeng Tian:** validation, writing – original draft, writing – review and editing. **Sisi Nie:** investigation, software, visualization. **Changmei Kou:** conceptualization, investigation, validation, visualization. **Meizu Wang:** data curation, software. **Shiyi Pu:** data curation, formal analysis, software, validation. **Zhihai Li:** project administration, resources.

## Funding

This work was supported by The Affiliated Anning First People's Hospital of Kunming University of Science and Technology under Grant KAZ2025001.

## Conflicts of Interest

The authors declare no conflicts of interest.

## Data Availability

The data that support the findings of this study are available from the corresponding author, Zhihai Li, upon reasonable request. The data are not publicly available due to privacy and ethical restrictions surrounding patient confidentiality, and are subject to the approval of the affiliated institution.
